# Relationships of Final Velocity at 30-15 Intermittent Fitness Test and Anaerobic Speed Reserve with Body Composition, Sprinting, Change-of-Direction and Vertical Jumping Performances: A Cross-Sectional Study in Youth Soccer Players

**DOI:** 10.3390/biology11020197

**Published:** 2022-01-27

**Authors:** Ana Filipa Silva, Sumer Alvurdu, Zeki Akyildiz, Filipe Manuel Clemente

**Affiliations:** 1 Escola Superior Desporto e Lazer, Instituto Politécnico de Viana do Castelo, Rua Escola Industrial e Comercial de Nun’Álvares, 4900-347 Viana do Castelo, Portugal; anafilsilva@gmail.com; 2 The Research Centre in Sports Sciences, Health Sciences and Human Development (CIDESD), 5001-801 Vila Real, Portugal; 3 Research Center in Sports Performance, Recreation, Innovation and Technology (SPRINT), 4960-320 Melgaço, Portugal; 4 Faculty of Sport Sciences, Gazi University, Ankara 06560, Turkey; sumeralvurdu@gazi.edu.tr (S.A.); zekiakyldz@hotmail.com (Z.A.); 5 Instituto de Telecomunicações, Delegação da Covilhã, 1049-001 Lisboa, Portugal

**Keywords:** football, athletic performance, physical fitness, exercise test, locomotor profile

## Abstract

**Simple Summary:**

Locomotor performance is constrained by how players generate and transmit force into the environment. Thus, understanding the factors that influence locomotor performance is important to optimizing players’ performance. The assessment can be performed while applying an intermittent progressive multistage running test. However, the performance of such tests depends on different physical qualities. Although many studies present relationships between performance in multistage tests and other physical qualities, there is a lack of evidence regarding the relationship of anaerobic speed reserve (difference between maximal sprint speed and velocity at maximal aerobic speed) with other physical variables. This may be relevant for identifying the factors that can optimize locomotor performance. Our study was conducted to identify the relationships of performance at a multistage test (30-15 Intermittent Fitness Test) and anaerobic speed reserve with the sprinting, change-of-direction, and neuromuscular capacities of youth soccer players. The findings of this research revealed that performance on an intermittent progressive multistage test is correlated with neuromuscular, sprinting, and change-of-direction abilities, while anaerobic speed reserve seems to be relatively independent of those qualities.

**Abstract:**

This study aimed to determine the relationships of locomotor profile (combination of a player’s maximal oxygen uptake and running economy)—measured by the final velocity attained at 30-15 Intermittent Fitness Test (V_IFT_) and the anaerobic speed reserve (ASR)—with the body composition, countermovement jump (CMJ), sprinting performances, and change-of-direction (COD) ability of youth soccer players. A cross-sectional study design was implemented. A total of 124 youth soccer players from different age groups (15, 16, 17, 18, and 19 years old) were voluntarily recruited. ASR was determined based on the difference between maximal sprint speed (MSS) and V_IFT_. Players were tested for the following measures: (i) body composition (body mass and body fat percentage); (ii) CMJ (height of jump); (iii) sprinting time measured at 5, 10, 15, 20, 25, and 30 m; (iv) MSS measured in the best split time (5 m) over the 30 m test; (v) COD ability (time, asymmetry index); and (vi) final velocity at 30-15 IFT (V_IFT_). A Pearson product-moment correlation test was used to examine the relationships. Height and body mass exhibited large correlations with V_IFT_ (r = 0.835 and r = 0.699, respectively) and small correlations with ASR (r = 0.177 and r = 0.256, respectively). The CMJ was largely correlated with V_IFT_ (r = 0.631 to r = 0.650) while presenting small correlations with ASR (r = 0.227 to r = 0.232). Both V_IFT_ and ASR had moderate (r = 0.3 to r = 0.5) correlations with sprinting time at different distances and very large correlations with MSS (r = 0.797 to r = 0.866). The COD time was largely correlated with V_IFT_ (r = 0.765 and r = 0.775) while exhibiting small-to-moderate correlations with ASR (r = −0.279 and r = −0.301). In conclusion, it was found that locomotor performance at 30-15 V_IFT_ presents high levels of correlation with anthropometry and physical fitness; ASR also presents correlations with these variables, but they are smaller. This suggest that ASR is an independent variable that should be considered for inclusion in information for characterizing players’ capacities.

## 1. Introduction

Soccer requires players to possess multiple well-developed physical qualities that support the physical demands that emerge from the match [1,2]. Over the regular time of a match, the predominance of oxidative energetic systems is evident, although anaerobic systems also play an important role during intensification moments [3]. Thus, soccer is intermittent and mixed from a bioenergetics point of view [4]. During a match, low-to-moderate running demands are predominant, although high-intensity demands such as high-intensity running, sprinting, accelerating, and decelerating can be critical during key moments (e.g., quick transitions, counterattacks) [5,6]. It is also important to highlight that over the last decade, matches have become more intense, as exemplified by studies conducted in the English and Spanish professional premier leagues [7,8,9] in which, for the same total distance covered in the matches, significant increases in the percentage of time is spent performing high-intensity running (in some cases, increases of up to 37% were observed) and sprinting (in some cases, increases up to 70% were observed).

Sustaining the intensity of the match is the target of the training process, and different training methods have focused on this criterion to develop the physical qualities of soccer players [10,11]. Even in the case of youth players, the approach to a more attuned training stimulus is one of the concerns of coaches [11,12]. Thus, possessing well-developed physical fitness is considered a key factor for sustaining physical demands and ultimately supporting the performance even in the case of youth [13,14]. Although playing position has a major effect on the relationship between physical fitness and match demands in youth players [15], physical qualities such as maximal speed in intermittent and progressive field-based tests seem to be highly associated with important measures such as total distance and high-intensity running covered in youth soccer matches [16]. The same has also been observed in professional men and women players, who present large to very-large correlations between maximal velocity achieved in a progressive field-based test and total distances and high-intensity distances covered in a match [17,18].

Since intermittent and progressive field-based tests can be of high value for determining the general fitness status of players, it is important to consider which physical qualities can concur to improve overall physical performance [19,20]. One well-known field-based test is the 30-15 Intermittent Fitness Test (30-15 IFT). This test is an intermittent progressive multistage test in which the pace increases by 0.5 km/h every 30 s, interspaced by 15 s of rest [21]. The main outcome of the 30-15 IFT is the final velocity at which the player cannot sustain the pace [21]. This test is commonly used for directly prescribing high-intensity interval training and can be associated with many physical qualities, thus providing more than a general idea of aerobic performance and an idea of the locomotor profile of a player [22].

The term “locomotor profile” suggests the relationship of cardiorespiratory measures with the locomotor demands attained during those measures, in which the maximal aerobic speed is the main outcome associated with the concept [23]. The final outcome in the 30-15 IFT (final velocity 30-15 IFT; V_IFT_) reflects maximal cardiorespiratory function, the anaerobic capacity [24], neuromuscular qualities [25], and inter-effort recovery abilities [26]. Thus, researchers [21,24,26,27] have reported the positive influence of change-of-direction (COD) ability, repeated sprint ability, lower limb power measured using countermovement jump (CMJ), and aerobic power on V_IFT_.

Although V_IFT_ can be used as a direct measure to standardize training intensity while applying high-intensity interval training, real standardization may not be attained [28]. In fact, for the same value of V_IFT_, the effort and intensity cannot be the same since the weight of intensity can be different for efforts above the V_IFT_. This is due to different ASRs that can be observed between players with the same V_IFT_ [28]. ASR represents the difference between maximal sprinting speed (MSS) and maximal aerobic speed (MAS). Although MAS is test dependent, the V_IFT_ can be understood as the specific MAS for the test (and not necessarily the same as the MAS measured under laboratory conditions) [22]. Thus, the combination of V_IFT_ (or MAS as in the original proposal) and MSS can provide an identification of the locomotor profile of a player, which can be classified as [28]: (i) speed profile (low V_IFT_/high MSS) with a large ASR; (ii) hybrid profile (moderate V_IFT_/moderate MSS) with a moderate ASR; and (iii) endurance profile (high V_IFT_/low MSS) with a small ASR. It is then expected that large ASR can be associated with fast-twitch fiber types, while moderate ASR can be associated with intermediate and small ASR with slow-twitch fiber types [28].

As previously described in the literature, V_IFT_ depends on CMJ (since it is an indirect outcome of lower limb power) and COD (since it is related to the mechanical aspects of turning) or sprinting [21,24,26,27]. Additionally, anthropometrics can play a role since body fat can influence performance. This knowledge about the relationships between V_IFT_ and physical qualities is well established and is not the same for ASR. ASR can be influenced by the maturation status, playing position, or running speed profile of youth soccer players [29,30]. Although a clear effect of speed profile and maximal aerobic speed for ASR has been found, to the best of our knowledge, the relationships of ASR with CMJ and COD abilities (e.g., deficit, asymmetry) have not been explored. Nevertheless, they are expected to be meaningful since they are indicators of a possible speed locomotor profile. Based on this hypothesis and considering the relevance of understanding the relationships between ASR and other physical qualities, the purpose of this study was to determine the relationships of ASR and V_IFT_ with body composition, sprinting performance, COD ability, and CMJ. Comparisons with tests with more than one trial will be performed considering all the trials executed and not the mean, since movement variability [31] can enhance particular changes in the results, thus biasing the final statistical values.

## 2. Materials and Methods

### 2.1. Study Design

This study followed a cross-sectional design. Convenience sampling was performed.

### 2.2. Setting

The five age groups included (14, 15, 16, 17, and 19 years old) were assessed on different days. The younger age group (14 years old) was assessed on 2 and 3 of September of 2021. The age groups of 15 and 16 years old were tested on 1 and 3 September of 2021. Finally, the older age groups (17 and 19 years old) were assessed on 1 and 2 of September of 2021. As a context, the assessments were performed in 3 weeks (24 training sessions and 3 non-official matches) after the beginning of the season.

### 2.3. Participants

The sample size was estimated a priori using G*Power (version 3.1.) with a magnitude of correlation of 0.3 (small), power of 0.80, and a significance of 0.05 which provided an estimated sample of 84 to determine significant levels. A total of 124 young male soccer players from 14 years old (*n* = 29), 15 years old (*n* = 30), 16 years old (*n* = 27), 17 years old (*n* = 25), and 18 years old (*n* = 12) youth teams of a professional team voluntarily participated in the study. During the regular season, these players trained twice a week (90 min per session) and played one match per week. The participants had to meet the following criteria: (i) be an active player with a three-year license, (ii) have no history of injuries in the previous two months, and (iii) attend 85 percent of the training sessions during the three weeks prior to the start of the assessments. Adult players and parents/legal guardians of youth players of the participants were informed about the main objectives and potential risks of the study, and signed informed consent forms. The study was conducted in accordance with the ethical principles of the Helsinki Declaration for human research and was approved by the Ethics Commission of the local university.

### 2.4. Testing Procedures

The assessments were performed after 2 days of recovery, considering the last training session. The tests were carried out on 2 different days with a 24 h rest intervals. Body composition measurements (height, body mass, and body fat percentage) and performance tests (vertical jumping, sprinting, and change-of-direction ability) were performed on the first day at 2:00 p.m. with a temperature of 24 degrees Celsius and 52% relative humidity. On the second day, 30-15 IFT tests were performed to assess final velocity (V_IFT_) and anaerobic speed reserve (ASR) under the following conditions: at 3:00 p.m., the temperature was 19 degrees Celsius with 49% relative humidity. Players were asked to have a regular dietary intake and no ingestion of stimulating drinks on the day of the assessments. A standardized FIFA 11+ warm-up protocol was applied before the performance tests [32] and all players were familiarized with the tests protocol and then were organized by groups.

### 2.5. Body Composition

The participants’ height and body mass were measured using a measuring tape (SECA 206, Hamburg, Germany) and a digital scale (SECA 874, Hamburg, Germany) with a 0.1 kg precision. The Durnin–Womersley formula [33] was used to calculate body fat percentage using four skinfold measurements (biceps, triceps, iliac crest, and subscapular). Each athlete had at least two measures taken, and if there was a difference of greater than 5% between the two, a third measurement was applied.

### 2.6. Jumping Performance

The Optojump optical measurement device (OptojumpNext, Microgate, Bolzano, Italy) was utilized to evaluate individuals’ jumping abilities using the Countermovement Jump (CMJ) test. The CMJ technique was demonstrated to participants, and after that, they had one non-recorded trial to ensure that the familiarization was correctly performed. The participants made three vertical attempts with a 2 min rest period in between, with the best attempt being considered for the analysis. The participants were instructed to jump from take-off to landing with their hands on their hips and without bending their legs.

### 2.7. Sprinting

The electronic timing gates system (Smartspeed, Fusion Sport, Brisbane, QLD, Australia) was used to measure the 30 m linear sprint test with 5 m intervals. The timing gates were installed at a height of 1.2 m above the floor. Players were positioned 0.5 m away from the first timing gate and encouraged to sprint as fast as they could. They were given two attempts with three minutes of rest to avoid fatigue. Before the start, players took one step forward with their preferred foot, with no signal given. For the analysis, the best sprinting time (lower value) was used.

### 2.8. Maximal Speed Sprint (MSS)

The MSS was considered in the current study as the highest running speed measured over the 30 m sprint test. The distance from 20 to 30 m seems to be enough in soccer players to determine the MSS [34]. In our case, the MSS was estimated using the average time over the last 10 and 5 m splits of a 30 m sprint test. A previous study revealed that using both 10 and 5 m splits of a 30 m sprint test while using timing gates can be reliable and present a high level of agreement with the MSS estimated using a gold-standard radar gun [35].

### 2.9. Change-of-Direction Ability

The subjects’ COD ability was assessed using the Arrowhead agility test. At the start line, an electronic timing gates system (Smartspeed, Fusion Sport, Brisbane, QLD, Australia) with a height of 1.2 m starting from the floor was installed. The participant sprinted from the start line to the middle marker (A), turned to the left or right side to sprint around the marker (B), sprinted around the top marker (C), then sprinted back through the timing gate to complete the test [36]. Athletes were instructed to use their right leg as a braking leg when turning left and their left leg as a braking leg when turning right. The test consisted of four randomized tries separated by at least three minutes of recovery time on both the left and right sides. Each side’s best efforts were recorded for analysis.

The following formula [37] was used to determine the percentage of asymmetry index (AI%):

AI% = [(COD time Dominant − COD time Non-dominant)/COD time Dominant] × 100

The dominant leg was defined as the plant foot (direction) with the fastest completion time following a previous methodological approach [37].

### 2.10. Velocity at 30-15 IFT and Anaerobic Speed Reserve

The participants completed the 30-15 IFT according to the procedure described by Buchheit [21]. Players completed 30 s of shuttle runs followed by 15 s of passive recovery to join the closest line from where they began the next stage from a standing position. The inability to match the covered distance with the auditory signal three times in a row was classified as exhaustion.

The final velocity (V_IFT_) was obtained from the last completed stage, and anaerobic speed reserve was estimated as the difference between MSS and V_IFT_ using the following equation [38]:

ASR (km/h) = MSS − V_IFT_ (1)

### 2.11. Statistical Analyses

The descriptive statistics were presented in form of mean, standard deviation, and coefficient of variation (%). The information in tables presents the data for trials 1 and 2, obtained in which tests in which more than 1 trial was conducted. Both trials were assessed in the same assessment day. The normality and homogeneity of the sample were inspected using the Kolmogorov–Smirnov and Levene’s test. After confirmation of the normality (*p* > 0.05) and homogeneity (*p* > 0.05) of the sample (for the different outcomes), the Pearson product-moment correlation test was conducted to examine the relationships of VIFT and ASR with the remaining physical fitness and anthropometric outcomes. The magnitude of correlations were considered based on the following thresholds [39,40,41]: trivial (0.0), small (0.1), medium (0.3), large (0.5), very large (0.7), almost perfect (0.9), and perfect (1.0). The CV was rated as good when CV < 5%, moderate when CV was 5–10% and poor when CV was >10% [42]. An alpha value of *p* < 0.05 was determined as the significance level [39]. All statistical analyzes were made using Rstudio version 1.3.1093.

## 3. Results

Table 1 presents the descriptive statistics of the outcomes analyzed in the current study. The values are the mean and standard deviation for the five age-groups assessed in the current study.

Table 2 presents the relationships of V_IFT_ and ASR with anthropometric outcomes. Very large correlations were observed between V_IFT_ and height (r = 0.835) and body mass (r = 0.699). Small correlations were observed between ASR and height (r = 0.177) and body mass (r = 0.256).

Table 3 presents the relationships of V_IFT_ and ASR with CMJ. Large correlations were observed between V_IFT_ and CMJ trials 1 (r = 0.650) and 2 (r = 0.631). Small correlations were observed between ASR and CMJ trials 1 (r = 0.232) and 2 (r = 0.227).

Table 4 presents the relationships between V_IFT_ and sprinting time and speed. Very large correlations were found between V_IFT_ and average speed at 20–25 m (r = 0.798), 25–30 m (r = 0.767) and maximal speed (r = 0.797).

Table 5 presents the relationships between ASR and sprinting time and speed. Very large correlations were found between ASR and sprinting time at 25–30 m (r = −0.802), average speed at 25–30 m (r = 0.830) and maximal sprinting speed (r = 0.866).

Table 6 presents the relationships between V_IFT_ and COD variables. Very large correlations were found between V_IFT_ and COD time average of both legs in trials 1 (r = 0.767) and 2 (r = 0.775), COD of dominant (r = 0.765) and non-dominant leg (r = 0.765).

Table 7 presents the relationships between ASR and COD variables. Moderate correlations were found between ASR and COD time average for both legs in trial 2 (r = −0.301).

## 4. Discussion

The relationship between V_IFT_ and ASR was tested with different physical fitness variables that are linked with the overall physical performance of youth soccer players. Large correlations between ASR and COD ability or CMJ were expected, considering the possible associations with greater fast-twitch fiber type. However, the correlations were small-to-moderate, and thus smaller than those observed in V_IFT_. As expected based on previous reports [22], V_IFT_ was largely correlated with height, body mass, COD ability, CMJ, and MSS. On the other hand, ASR was largely correlated with MSS.

The main purpose of the current study was to determine the association between ASR and different physical qualities. Considering that ASR represents a simple difference between MSS and the MAS, it is likely that some power-related variables contribute to or are associated with ASR. However, according to the parameters analyzed (which included anthropometry, CMJ, sprinting performance, and COD performance), the only large correlations were with MSS. Although significant correlations with other physical parameters were detected, they were not large.

MSS itself is part of the equation used to determine ASR; thus, a correlation of such a magnitude is expected. Interestingly, these results are in line with previous reports [30,38] that confirm the relationship between ASR and absolute locomotor profile. Naturally, ASR can be associated with faster players that achieve higher MSS while not having the best MAS, which creates a greater ASR [30]. Those cases justify why faster players can also hold higher ASR, thus being in the line of “speed profile” [28].

However, small correlations with many other physical qualities measured in this experiment were not expected. Possibly, ASR is a relatively isolated measure that should be considered part of a player’s locomotor profile. For example, in a study considering the maturation effect on the relationships between repeated sprint ability and ASR, it was obvious that the measures become independent after peak height velocity is reached [29]. Thus, ASR cannot be highly dependent on regular physical qualities, and the metabolic profile can be part of the equation when looking into relationships. This provides value to ASR. In fact, in the case of exercise, lower percentages of ASR are associated with greater exercise capacity [28]. Thus, this measure can be of great interest for deciding which measures to include and use for training.

Regarding V_IFT_, this measure was highly associated with anthropometric measures, CMJ, sprinting performance, and COD performance. This is in line with the use of V_IFT_ as a composite measure [22]. For example, a high level of correlation between V_IFT_ and COD is expected since the mechanical demands presented while turning and the fastest COD can support players during the final stages of the 30-15 IFT test [22]. Additionally, the stretching–shortening cycle presented in CMJ, as well as the muscle power represented by the vertical jump, can be relevant for explaining the attainment of the highest efforts in the latest stages of the 30-15 IFT. Thus, as illustrated by the proponents of the 30-15 IFT [22], in the worst-case scenario, the test can be used as an overall and composite overview of the physical fitness status of players since it depends on multiple different physical qualities.

This study had some limitations. For instance, the maturation stage was not determined for those closer to peak height velocity (under-15); thus, it is a possible concurrent factor influencing the final outcomes. Future studies should include a marker of maturation to act as a moderator for the results. Additionally, future studies should focus on gaining additional information about fiber types and the metabolic profiles of players and determine how these factors interact to justify ASRs. This can be a determinant for prescribing training based on ASR and targeting the players based on their profiles.

Despite its limitations, this study is one of the very few that tested the relationships between physical fitness parameters and ASR in youth soccer players from different age groups. As practical implications, we can attest to the idea that V_IFT_ can represent multiple physical qualities that can be used by coaches to obtain a brief overview of the physical fitness levels of players. On the other hand, this study suggests that ASR is an isolated measure that depends to some extent on other physical qualities, thus attesting to the importance of classifying players’ profiles.

## 5. Conclusions

The current cross-sectional study revealed that both V_IFT_ and ASR are largely correlated with maximal speed sprint. However, V_IFT_ had greater correlations with anthropometry, COD ability, and CMJ than ASR did. This suggests that V_IFT_ better represents multiple dependencies on different physical qualities, while ASR is a more independent and isolated measure that can be considered as a physical measure itself that can also be used to classify players. As a practical implication, this study indicates that oriented training focusing on improving neuromuscular performance can offer additional contributions for improving players’ locomotor profiles (besides the natural metabolic work).

## Figures and Tables

**Table 1:** Descriptive statistics (mean and standard deviation) of the outcomes.

Values	14 yo	15 yo	16 yo	17 yo	18 yo	Overall
Height (cm)	167.65 ± 7.02 CV%: 4.2	174.80 ± 4.70 CV%: 2.7	176.14 ± 6.63 CV%: 3.8	175.48 ± 7.04 CV%: 4	177.16 ± 4.89 CV%: 2.8	173.78 ± 7.07 CV%: 4.1
Body Mass (kg)	57.43 ± 7.90 CV%: 13.8	64.540 ± 5.79 CV%: 9	65.822 ± 5.96 CV%: 9.1	67.780 ± 6.52 CV%: 9.6	69.325 ± 5.77 CV%: 8.3	64.27 ± 7.61 CV%: 11.8
Body Fat (%)	8.94 ± 2.82 CV%: 31.6	7.82 ± 1.69 CV%: 21.6	8.14 ± 1.81 CV%: 22.2	9.06 ± 2.30 CV%: 25.4	8.45 ± 1.93 CV%: 22.9	8.47 ± 2.20 CV%: 26
CMJ—trial 1 (cm)	36.95 ± 4.70 CV%: 12.7	37.96 ± 4.49 CV%: 11.8	42.08 ± 7.07 CV%: 16.8	42.02 ± 6.04 CV%: 14.4	41.04 ± 4.92 CV%: 12	39.75 ± 5.91 CV%: 14.9
CMJ—trial 2 (cm)	35.70 ± 5.13 CV%: 14.4	36.37 ± 4.53 CV%: 12.5	40.45 ± 6.94 CV%: 17.2	40.92 ± 6.24 CV%: 15.3	39.81 ± 5.04 CV%: 12.7	38.37 ± 6.02 CV%: 15.7
Sprint time at 5 m—trial 1 (s)	1.39 ± 0.10 CV%: 7.4	1.29 ± 0.11 CV%: 9.2	1.29 ± 0.09 CV%: 7.5	1.10 ± 0.11 CV%: 10.4	1.10 ± 0.08 CV%: 7.9	1.26 ± 0.15 CV%: 12
Sprint time at 5 m—trial 2 (s)	1.46 ± 0.18 CV%: 12.3	1.42 ± 0.16 CV%: 11.2	1.40 ± 0.14 CV%: 10.5	1.19 ± 0.13 CV%: 11.3	1.25 ± 0.12 CV%: 9.8	1.36 ± 0.18 CV%: 13.5
Sprint time at 10 m—trial 1 (s)	2.14 ± 0.11 CV%: 5.2	2.04 ± 0.11 CV%: 5.7	2.04 ± 0.09 CV%: 4.7	1.81 ± 0.14 CV%: 8.2	1.79 ± 0.10 CV%: 5.9	1.99 ± 0.17 CV%: 8.8
Sprint time at 10 m—trial 2 (s)	2.21 ± 0.18 CV%: 8.5	2.17 ± 0.16 CV%: 7.5	2.17 ± 0.14 CV%: 6.9	1.92 ± 0.16 CV%: 8.5	1.94 ± 0.11 CV%: 5.7	2.10 ± 0.20 CV%: 9.5
Sprint time at 15 m—trial 1 (s)	2.88 ± 0.14 CV%: 4.9	2.70 ± 0.15 CV%: 5.6	2.68 ± 0.11 CV%: 4.4	2.44 ± 0.19 CV%: 7.9	2.41 ± 0.12 CV%: 5	2.66 ± 0.22 CV%: 8.4
Sprint time at 15 m—trial 2 (s)	2.95 ± 0.20 CV%: 6.9	2.84 ± 0.17 CV%: 6.1	2.78 ± 0.16 CV%: 6	2.50 ± 0.26 CV%: 10.6	2.46 ± 0.30 CV%: 12.4	2.75 ± 0.27 CV%: 8.4
Sprint time at 20 m—trial 1 (s)	3.57 ± 0.16 CV%: 4.6	3.38 ± 0.16 CV%: 5	3.32 ± 0.14 CV%: 4.2	3.07 ± 0.20 CV%: 6.7	3.03 ± 0.12 CV%: 4	3.31 ± 0.25 CV%: 7.6
Sprint time at 20 m—trial 2 (s)	3.64 ± 0.21 CV%: 5.9	3.51 ± 0.18 CV%: 5.3	3.44 ± 0.14 CV%: 4.3	3.16 ± 0.22 CV%: 6.9	3.20 ± 0.12 CV%: 3.8	3.42 ± 0.25 CV%: 7.5
Sprint time at 25 m—trial 1 (s)	4.21 ± 0.19 CV%: 4.6	4.06 ± 0.17 CV%: 4.4	3.97 ± 0.16 CV%: 4	3.68 ± 0.22 CV%: 6.2	3.63 ± 0.14 CV%: 3.9	3.96 ± 0.28 CV%: 7.1
Sprint time at 25 m—trial 2 (s)	4.29 ± 0.22 CV%: 5.3	4.20 ± 0.18 CV%: 4.5	4.09 ± 0.16 CV%: 3.9	3.78 ± 0.24 CV%: 6.4	3.79 ± 0.14 CV%: 3.7	4.07 ± 0.28 CV%: 6.9
Sprint time at 30 m—trial 1 (s)	4.86 ± 0.22 CV%: 4.6	4.65 ± 0.20 CV%: 4.5	4.54 ± 0.18 CV%: 4.1	4.24 ± 0.23 CV%: 5.5	4.21 ± 0.16 CV%: 3.8	4.55 ± 0.31 CV%: 6.9
Sprint time at 30 m—trial 2 (s)	4.94 ± 0.25 CV%: 5.1	4.79 ± 0.22 CV%: 4.6	4.67 ± 0.19 CV%: 4.1	4.34 ± 0.24 CV%: 5.7	4.37 ± 0.15 CV%: 3.5	4.67 ± 0.31 CV%: 6.8
Sprint time at 20–25 m—trial 1 (s)	0.64 ± 0.03 CV%: 5.9	0.68 ± 0..04 CV%: 6.9	0.64 ± 0.05 CV%: 9.1	0.61 ± 0.03 CV%: 5.6	0.59 ± 0.03 CV%: 5.7	0.64 ± 0.05 CV%: 8.2
Sprint time at 25–30 m—trial 2 (s)	0.64 ± 0.04 CV%: 7.5	0.59 ± 0.05 CV%: 9.8	0.57 ± 0.06 CV%: 11	0.56 ± 0.04 CV%: 7.5	0.57 ± 0.05 CV%: 9	0.59 ± 0.06 CV%: 10.2
Average speed at 20–25 m (km/h)	27.83 ± 1.67 CV%: 6	26.56 ± 1.80 CV%: 6.8	27.99 ± 2.17 CV%: 7.8	29.58 ± 1.66 CV%: 5.6	30.43 ± 1.69 CV%: 5.6	28.16 ± 2.19 CV%: 7.8
Average speed at 25–30 m (km/h)	27.94 ± 2.04 CV%: 7.3	30.65 ± 2.93 CV%: 9.6	31.47 ± 3.25 CV%: 10.3	32.01 ± 2.43 CV%: 7.6	31.31 ± 2.62 CV%: 8.4	30.53 ± 3.05 CV%: 10
Max speed (km/h)	28.60 ± 1.69 CV%: 5.9	30.80 ± 2.72 CV%: 8.8	31.85 ± 2.75 CV%: 8.6	32.24 ± 0.221 CV%: 6.9	32.01 ± 1.88 CV%: 5.9	30.92 ± 2.69 CV%: 8.7
COD Right leg—trial 1 (s)	9.24 ± 0.25 CV%: 2.8	9 ± 0.36 CV%: 4	8.90 ± 0.29 CV%: 3.3	8.71 ± 0.22 CV%: 2.6	8.70 ± 0.14 CV%: 1.7	8.95 ± 0.34 CV%: 3.8
COD Right leg—trial 2 (s)	9.36 ± 0.26 CV%: 2.8	9.12 ± 0.37 CV%: 4.1	9.02 ± 0.31 CV%: 3.5	8.87 ± 0.21 CV%: 2.4	8.83 ± 0.17 CV%: 1.9	9.08 ± 0.34 CV%: 3.8
COD Left leg—trial 1 (s)	9.20 ± 0.29 CV%: 3.2	8.97 ± 0.31 CV%: 3.6	8.88 ± 0.29 CV%: 3.3	8.76 ± 0.25 CV%: 2.9	8.67 ± 0.18 CV%: 2.1	8.93 ± 0.33 CV%: 3.7
COD Left leg—trial 2 (s)	9.32 ± 0.29 CV%: 3.1	9.09 ± 0.31 CV%: 3.5	9.01 ± 0.29 CV%: 3.3	8.92 ± 0.29 CV%: 3.3	8.79 ± 0.20 CV%: 2.4	9.06 ± 0.33 CV%: 3.7
COD Average both legs—trial 1 (s)	9.23 ± 0.25 CV%: 2.8	8.99 ± 0.30 CV%: 3.4	8.89 ± 0.28 CV%: 3.2	8.74 ± 0.22 CV%: 2.6	8.69 ± 0.13 CV%: 1.6	8.94 ± 0.31 CV%: 3.6
COD Average both legs—trial 2 (s)	9.34 ± 0.25 CV%: 2.7	9.11 ± 0.31 CV%: 3.4	9.02 ± 0.29 CV%: 3.2	8.90 ± 0.23 CV%: 2.7	8.82 ± 0.16 CV%: 1.8	9.07 ± 0.31 CV%: 3.5
COD Dominant leg (s)	9.13 ± 0.24 CV%: 2.7	8.87 ± 0.34 CV%: 3.9	8.83 ± 0.29 CV%: 3.3	8.66 ± 0.23 CV%: 2.7	8.61 ± 0.15 CV%: 1.8	8.86 ± 0.32 CV%: 3.7
COD Non-dominant leg (s)	9.31 ± 0.27 CV%: 2.9	9.10 ± 0.29 CV%: 3.2	8.95 ± 0.28 CV%: 3.2	8.81 ± 0.22 CV%: 2.6	8.76 ± 0.15 CV%: 1.7	9.02 ± 0.32 CV%: 3.6
COD Asymmetry index (A.U.)	−1.94 ± 1.19 CV%: −61	−2.64 ± 2.50 CV%: −94	−1.34 ± 0.76 CV%: −56	−1.74 ± 0.89 CV%: −51	−1.64 ± 1.35 CV%: −82	−1.91 ± 1.57 CV%: −82
V_IFT_ (km/h)	17.44 ± 1.49 CV%: 8.6	18.35 ± 1.19 CV%: 6.5	18.44 ± 1.08 CV%: 5.9	18.80 ± 1.24 CV%: 6.6	19.54 ± 0.89 CV%: 4.6	18.36 ± 1.36 CV%: 7.4
ASR (km/h)	11.15 ± 2.05 CV%: 18.4	12.45 ± 3.04 CV%: 24.4	13.41 ± 2.80 CV%: 20.9	13.44 ± 2.28 CV%: 17	12.47 ± 2.30 CV%: 18.5	12.56 ± 2.67 CV%: 21.3

yo: years old; CMJ: countermovement jump; COD: change-of-direction; V_IFT_: final velocity at 30-15 Intermittent Fitness Test; ASR: anaerobic speed reserve; A.U.: arbitrary units; CV: coefficient of variation; SD: standard deviation.

**Table 2:** Relationship (Pearson correlation coefficient) between V_IFT_ and ASR and anthropometry variables.

	V_IFT_ and H	V_IFT_ and BM	V_IFT_ and BF	ASR and H	ASR and BM	ASR and BF
r (average)	0.835 ***	0.699 ***	−0.112	0.177 *	0.256 **	−0.039
r (95%CI: max)	0.881	0.778	0.063	0.343	0.413	0.139
r (95%CI: min)	0.773	0.598	−0.280	0.000	0.083	−0.214

* *p* < 0.05, ** *p* < 0.01, *** *p* < 0.001; H: height; BM: body mass; BF: body fat; V_IFT_: final velocity at 30-15 Intermittent Fitness Test; ASR: anaerobic speed reserve.

**Table 3:** Relationship (Pearson correlation coefficient) between V_IFT_ and ASR and CMJ variables.

	V_IFT_ and CMJ Trial 1	V_IFT_ and CMJ Trial 2	ASR and CMJ Trial 1	ASR and CMJ Trial 2
r (average)	0.650 ***	0.631 **	0.232 **	0.227 *
r (95%CI: max)	0.740	0.725	0.392	0.387
r (95%CI: min)	0.537	0.514	0.058	0.052

* *p* < 0.05, ** *p* < 0.01, *** *p* < 0.001; CMJ: counter movement jump; V_IFT_: final velocity at 30-15 Intermittent Fitness Test; ASR: anaerobic speed reserve.

**Table 4:** Relationship (Pearson correlation coefficient) between V_IFT_ and sprinting time and speed variables.

**Outcomes**	**V_IFT_ and 5 m Trial 1**	**V_IFT_ and 5 m Trial 2**	**V_IFT_ and 10 m Trial 1**	**V_IFT_ and 10 m Trial 2**	**V_IFT_ and 15 m Trial 1**	**V_IFT_ and 15 m Trial 2**	**V_IFT_ and 20 m Trial 1**	**V_IFT_ and 20 m Trial 2**	**V_IFT_ and 25 m Trial 1**	**V_IFT_ and 25 m Trial 2**
r (average)	−0.365 ***	−0.365 ***	−0.216 *	−0.220 *	−0.093	−0.163	0.098	0.129	0.267 **	0.305 **
r (95%CI: max)	−0.205	−0.204	−0.044	−0.048	0.082	−0.011	0.267	0.296	0.421	0.455
r (95%CI: min)	−0.507	−0.506	−0.375	−0.379	−0.262	−0.327	−0.077	−0.046	0.097	0.139
**Outcomes**	**V_IFT_ and 30 m Trial 1**	**V_IFT_ and 30 m Trial 2**	**V_IFT_ and 20–25 m**	**V_IFT_ and 25–30**	**VI_FT_ and Average Speed at 20–25**	**V_IFT_ and Average Speed at 25–30**	**V_IFT_ and Maximal Sprint Speed**			
r (average)	0.368 **	0.402 **	−0.379 **	−0.404 ***	0.798 **	0.767 **	0.797 **			
r (95%CI: max)	0.509	0.538	−0.219	−0.248	0.854	0.830	0.852			
r (95%CI: min)	0.208	0.246	−0.518	−0.540	0.725	0.685	0.723			

* *p* < 0.05, ** *p* < 0.01, *** *p* < 0.001; V_IFT_: final velocity at 30-15 intermittent fitness test.

**Table 5:** Relationship (Pearson correlation coefficient) between ASR and sprinting time and speed variables.

**Outcome**	**ASR and 5 m Trial 1**	**ASR and 5 m Trial 2**	**ASR and 10 m Trial 1**	**ASR and 10 m Trial 2**	**ASR and 15 m Trial 1**	**ASR and 15 m Trial 2**	**ASR and 20 m Trial 1**	**ASR and 20 m Trial 2**	**ASR and 25 m Trial 1**	**ASR and 25 m Trial 2**
r (average)	−0.358 ***	−0.258 **	−0.311 ***	−0.227 ***	−0.349 ***	−0.251 **	−0.369 ***	−0.324 ***	−0.333 ***	−0.293
r (95%CI: max)	−0.194	−0.086	−0.142	−0.052	−0.184	−0.078	−0.206	−0.156	−0.166	−0.123
r (95%CI: min)	−0.503	−0.416	−0.462	−0.387	−0.495	−0.409	−0.512	−0.473	−0.481	−0.446
**Outcome**	**ASR and 30 m Trial 1**	**ASR and 30 m Trial 2**	**ASR and 20−25 m**	**ASR and 25−30**	**ASR and Average Speed at 20−25**	**ASR and Average Speed at 25−30**	**ASR and Maximal Sprint Speed**			
r (average)	−0.451 ***	−0.417 ***	−0.017	−0.802 ***	0.055	0.830 ***	0.866 ***			
r (95%CI: max)	−0.299	−0.260	0.160	−0.728	0.229	0.878	0.904			
r (95%CI: min)	−0.581	−0.553	−0.193	−0.857	−0.123	0.765	0.814			

** *p* < 0.01, *** *p* < 0.001; ASR: anaerobic speed reserve.

**Table 6:** Relationship (Pearson correlation coefficient) between V_IFT_ and COD variables.

	V_IFT_ and COD Right Trial 1	V_IFT_ and COD Right Trial 2	V_IFT_ and COD Left Trial 1	V_IFT_ and COD Left Trial 2	V_IFT_ and COD Average Both Legs Trial 1	V_IFT_ and COD Average Both Legs Trial 2	V_IFT_ and Dominante	V_IFT_ and Non-Dominante	V_IFT_ and AsymetryIndex
r (average)	0.761 ***	0.769 ***	0.764 ***	0.771 ***	0.767 ***	0.775 ***	0.765 ***	0.765 ***	0.409 ***
r (95%CI: max)	0.826	0.831	0.828	0.833	0.830	0.836	0.829	0.829	0.544
r (95%CI: min)	0.677	0.687	0.680	0.690	0.684	0.694	0.682	0.682	0.254

*** *p* < 0.001; V_IFT_: final velocity at 30-15 Intermittent Fitness Test; COD: change-of-direction.

**Table 7:** Relationship (Pearson correlation coefficient) between ASR and COD variables.

	ASR and COD Right Trial 1	ASR and COD Right Trial 2	ASR and COD Left Trial 1	ASR and COD Left Trial 2	ASR and COD Average Both Legs Trial 1	ASR and COD Average Both Legs Trial 2	ASR and Dominante	ASR and Non-Dominante	ASR * AsymetryIndex
r (average)	−0.240 **	−0.256 **	−0.290 **	−0.307 ***	−0.279 **	−0.301 ***	−0.251 **	−0.295 ***	0.096
r (95%CI: max)	−0.066	−0.083	−0.120	−0.138	−0.108	−0.132	−0.079	−0.125	0.265
r (95%CI: min)	−0.399	−0.414	−0.443	−0.458	−0.434	−0.453	−0.410	−0.448	−0.082

* *p* < 0.05, ** *p* < 0.01, *** *p* < 0.001; ASR: anaerobic speed reserve; COD: change-of-direction.

## Data Availability

Not applicable.
